# Dementia Improvement after Primary Hyperparathyroidism Surgery

**DOI:** 10.1155/2020/1095414

**Published:** 2020-06-24

**Authors:** Claudio Voci

**Affiliations:** Azienda Ospedaliero‐Universitaria of Bologna, Bologna, Italy

## Abstract

This is the case of a 76-year-old man admitted to hospital in a delirium state, previously diagnosed with a major depressive disorder at an age of 50 years, treated for years for chronic tension headache. The computed tomography of the head resulted negative. Inpatient laboratory tests revealed a mild hypercalcemia. Due to the progression of the disease (delirium state, dementia, tension headache, and depression), he was again admitted to hospital. The patient showed dysarthria, postural tremors, mirror movements and palmar hyperhidrosis, mild ataxia when walking, and rigidity. Sleep disturbances were also observed. He underwent several clinical diagnostic tests, which resulted negative. After more than 2 years, the ultrasound of the neck identified enlarged parathyroid glands. The patient was surgically treated, and three parathyroid glands were removed. Parathyroidectomy and lithium treatment resulted in improvement of cognitive functions. In elderly patients, concomitant presence of cognitive dysfunction may mask the underlying primary hyperparathyroidism.

## 1. Background

The prevalence of patients with primary hyperparathyroidism (PHPT) is 0.4% and 10 times more in elderly people [1]. In PHPT, one or more of the parathyroid glands is overactive that causes calcium blood levels to rise [2]. Due to the elevated calcium levels, patients typically present with the classic bone and kidney disease. Among older patients, PHPT may manifest as various neuropsychiatric symptoms, ranging from affective disorders, general apathy, anxiety, sleep disorders, personality changes and cognitive impairment, and dementia to more severe psychotic conditions.

The main causes of PHPT are noncancerous growth (adenoma) on a gland (the most common cause, 75 to 85% of cases); an enlargement (hyperplasia) of two or more parathyroid glands (4 to 5%); and rarely, a cancerous (malignant) tumor (1%) [3].

In case of symptomatic PHPT, guidelines suggest surgery as the first treatment option. This includes those patients with substantially elevated calcium levels, as well as those with signs or symptoms of kidney stones or bone disease.

About 75% of people with PHPT have no symptoms. The problem is often picked up incidentally on routine blood sampling for other reasons via raised calcium (Ca) and parathyroid hormone (PTH) levels.

In case of asymptomatic disease, monitoring symptoms and control of biochemical parameters are recommended. Parathyroidectomy is appropriate among younger patients and patients at the risk of progression to symptomatic disease.

Some studies showed improvement in the neurocognitive function after parathyroidectomy [4, 5]. That suggests a vigilant search of PHTP among elderly patients.

This is the case of a 76-year-old man with PHTP presenting as dementia, which reversed after parathyroidectomy.

## 2. Case Report

A 76-year-old Italian male was admitted to hospital in February 2017 in a delirium state. He had been diagnosed with a major depressive disorder at 50 years of age, and he has been treated for years for chronic tension headache with antidepressants, anxiolytics, benzodiazepines, and analgesics. He was discharged the same day with a diagnosis of delirium. The computed tomography (CT) of the head resulted negative. Inpatient laboratory tests revealed a mild hypercalcemia (Ca 11.4 mg/dl). Thus, the patient was suggested to undergo Ca level and PTH level tests during the following days, which revealed a Ca value of 10.6 mg/dl and a PTH value of 94 pg/mL.

Due to the progression of the disease (delirium state, dementia, tension headache, and depression), he was again admitted in March 2017. The patient, although being in his conscious level, showed dysarthria, postural tremors, mirror movements and palmar hyperhidrosis, mild ataxia when walking, and rigidity. Sleep disturbances were also observed. So, he was suggested to temporarily stop taking antidepressants, anxiolytics, and benzodiazepines, with no evident improvement.

As part of the differential diagnosis, he underwent several clinical diagnostic tests, which resulted negative: bone marrow test, tau protein and 14.3 dosage, electroencephalography, thyroid ultrasound, CT scan of the neck and abdomen, CT scan and magnetic resonance imaging of the head, and whole-body 18 FDG Positron Emission Tomography/Computed Tomography (PET/CT) that shows no pathological hypermetabolic activity. The result of the mini mental state examination (MMSE) was 12/30. The level of total Ca was 11.0 mg/dl, and the PTH level was 92 pg/mL. He was administered Immunoglobulin G (IgG) EV, 30 mg/day for 5 days. Insomnia was treated with cetirizine 20 mg/die. The discharge diagnosis was degenerative disease.

In July 2017, the endocrinology department was consulted to study the calcium metabolism and determined a hypothetical normocalcemic PHPT, associated with a vitamin D insufficiency. He was requested a vertebral and femoral bone density test, a thyroid ultrasound, and a parathyroid scintigraphy and prescribed vitamin D 25,000 IU daily for 4 weeks to increase the vitamin D level and better understand laboratoristic conditions. Then, he was suggested to repeat the Ca and PTH tests. At this time, the MMSE was 21/30. The thyroid ultrasound showed a suspicious image near the inferior margin of the left thyroid lobe consistent with a parathyroid of an increased size. The Ca level was 10.8 mg/dL, and PTH level was 91 pg/mL. Anxiety and depression still continued.

**Figure 1:**
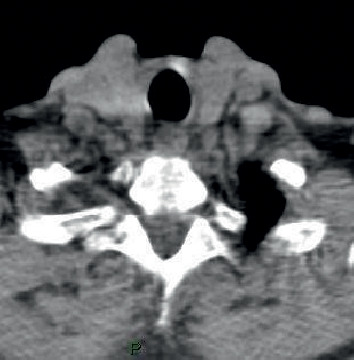
A 76-year-old male. The patient with dementia underwent 18F-FCH PET/CT for evaluation of parathyroid adenoma. The transaxial scan image of the neck did not show any abnormalities.

**Figure 2:**
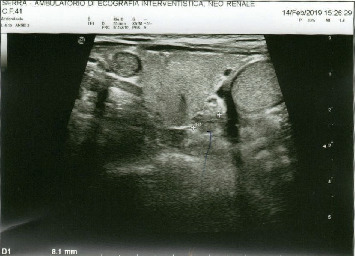
Grey scale sonographic image of the neck identified enlarged parathyroid glands (BLUE ARROW,dimension 11 × 8) on the left thyroid region.

In April 2018, he underwent CT scan, magnetic resonance tomography scan, and again, PET/CT, which were all negatives (Figure 1), and the ultrasound of the neck, which identified enlarged parathyroid glands (dimension 11 × 8; Figure 2.). The routine laboratory test showed a Ca level of 10.8 mg/dL. In October 2018, the Ca level was 10.9 mg/dL, Thyroidstimulating hormone (TSH) was 1.70 microU/mL, PTH was 97 pg/mL, and Thyroid peroxidase (TPO) antibodies were <1.0 UI/mL.

In January 2019, the Ca level increased to 11.4 mg/dL.

The endocrinology suggested, then, the parathyroidectomy, which removed 3 of 4 parathyroid glands. The parathyroid glands' biopsy showed hyperplasia with no signs of malignancy. During the first postsurgical week, the Ca level was 7 mg/dL and increased to 8.5 mg/dL due to the administration of calcium treatment. The thyroid function was restored, and in the subsequent weeks, the patient showed significant signs of recovery of cognitive impairment. In the follow-up (6 months), the MMSE was 25/30.

As there was still evidence of headaches, depression, and difficulties in sleeping and the Ca levels corrected, he was suggested to treat with oral lithium. After two months of therapy, these symptoms improved considerably.

## 3. Discussion

PHPT is a condition caused by an excessive and uncontrolled secretion of the parathyroid hormone. The classical presenting symptoms are stone and bone disease. Dysfunction of the central nervous system, peripheral nerve, and muscles could also occur in symptomatic patients [6, 7].

In the presented case, the classical symptoms were absent, and clinical picture at presentation was a delirium state, dementia, tension headache, and depression. Due to the poor cognitive and behavioural status, laboratory and imaging investigations were periodically performed. When the Ca levels increased and a thyroid ultrasound scan showed an image consistence with an increased parathyroid gland size, the surgical treatment with parathyroidectomy was decided. A remarkable improvement after parathyroidectomy at all levels was observed: movement, memory, attention, concentration, and general quality of life.

Finally, it was reported a better use of a cell phone [8].

Previous studies showed that surgical treatment appears to improve cognitive symptoms in patients with PHPT [9, 10]. Actually, this matter is controversial, and there is not a ful agreement.

According to the guidelines of the National Institutes of Health (NIH) for the management of asymptomatic PHPT [11], neurocognitive factors should be taken into account to identify the diagnosis and treatment of PHPT, but should not constitute the sole indication for surgery. Moreover, previous studies on the effect of surgery on cognitive symptoms in patients with PHPT showed that there was no significant improvement in cognitive deficits after successful parathyroidectomy.

In the present case, the remarkable improvement after parathyroidectomy at all levels may suggest early parathyroidectomy in case of PHPT with significant neurocognitive disturbances even in the absence of other symptoms of PHPT. In addition, this case highlights that, although rare, the underlying undiagnosed asymptomatic PHPT should be considered as a possible cause of dementia, even though asymptomatic PHPT presenting as dementia was reported rarely in the literature [12, 13].

Anyway, further research regarding the possible relationship between dementia associated with cognitive decline and undiagnosed PHPT is needed.

Until more definite evidence becomes available, for asymptomatic PHPT patients who are not eligible to surgery, guidelines suggest a long-term monitoring that consists in testing Ca values periodically and imaging investigations when appropriated.

Dementia, depression, and cognitive impairment are common conditions in several geriatric cases, and they could progress over time. PHPT, though common among the geriatric population, may be missed, and the symptoms may be prematurely diagnosed as cognitive impairments [14]. Since the important characteristic feature of hyperparathyroidism is the reversal of symptoms [15] by medical and surgical interventions, an early detection of all possible factors contributing to dementia is crucial to improve the quality of life of patients [16].

## Supplementary Materials

Supplementary Materials The supplementary material contains the “Care Checklist.”

## References

[1] Zini M., Attanasio R., Cesareo R (2012). AME position statement: primary hyperparathyroidism in clinical practice. *Journal of Endocrinological Investigation*.

[2] Gasser R. W. (2013). Clinical aspects of primary hyperparathyroidism: clinical manifestations, diagnosis, and therapy. *Wiener Medizinische Wochenschrift*.

[3] Ohya Y., Osaki M., Sakai S (2018). A case of hyperparathyroidism-associated parkinsonism successfully treated with cinacalcet hydrochloride, a calcimimetic. *BMC Neurology*.

[4] Khan A., Grey A., Shoback D. (2009). Medical management of asymptomatic primary hyperparathyroidism: proceedings of the third international workshop. *The Journal of Clinical Endocrinology & Metabolism*.

[5] Udelsman R., Pasieka J. L., Sturgeon C., Young J. E. M., Clark O. H. (2009). Surgery for asymptomatic primary hyperparathyroidism: proceedings of the third international workshop. *The Journal of Clinical Endocrinology & Metabolism*.

[6] Geffken G. R., Ward H. E., Staab J. P., Carmichael S. L. K., Evans D. L. (1998). Psychiatric morbidity in endocrine disorders. *Psychiatric Clinics of North America*.

[7] Roman S., Sosa J. A. (2007). Psychiatric and cognitive aspects of primary hyperparathyroidism. *Current Opinion in Oncology*.

[8] Levine D. M., Lipsitz S. R., Linder J. A. (2018). Changes in everyday and digital health technology use among seniors in declining health. *The Journals of Gerontology: Series A*.

[9] Roman S. A., Sosa J. A., Mayes L. (2005). Parathyroidectomy improves neurocognitive deficits in patients with primary hyperparathyroidism. *Surgery*.

[10] Roman S., Sosa J. A. (2007). Psychiatric and cognitive aspects of primary hyperparathyroidism. *Current Opinion in Oncology*.

[11] Bilezikian J. P., Brandi M. L., Eastell R. (2014 Oct). Guidelines for the management of asymptomatic primary hyperparathyroidism: summary statement from the fourth international workshop. *The Journal of Clinical Endocrinology & Metabolism*.

[12] Molaschi M., Ponzetto M., Romin R. (1994). Neuropsychologic symptoms of primary hyperparathyroidism in the elderly. Report of a clinical case. *Minerva Endocrinologica*.

[13] Joborn C., Hetta J., Frisk P., Palmer M., Akerstrom G., Ljunghall S. (1986). Primary hyperparathyroidism in patients with organic brain syndrome. *Acta Medica Scandinavia*.

[14] van Crevel H., van Gool W. A., Walstra G. J. M. (1999). Early diagnosis of dementia: which tests are indicated? what are their costs?. *Journal of Neurology*.

[15] Formiga F., Mascaró J., Chivite D., Pujol R. (2000). Reversible dementia due to two coexisting diseases. *The Lancet*.

[16] Flicker L., Ames D. (2005). Metabolic and endocrinological causes of dementia. *International Psychogeriatrics*.

